# Multimodal imaging of right ventricular obstruction due to metastatic cardiac tumour

**DOI:** 10.1093/ehjcr/ytab290

**Published:** 2021-07-24

**Authors:** Yuta Torii, Taiji Okada, Mariko Tokiwa, Yutaka Furukawa

**Affiliations:** 1 Department of Clinical Laboratory, Kobe City Medical Center General Hospital, 2-1-1 Minatojima Minamimachi, Chuo-ku, Kobe 650-0047, Hyogo, Japan; 2 Department of Cardiovascular Medicine, Kobe City Medical Center General Hospital, 2-1-1 Minatojima Minamimachi, Chuo-ku, Kobe 650-0047, Hyogo, Japan; 3 Department of Breast Surgery, Kobe City Medical Center General Hospital, 2-1-1 Minatojima Minamimachi, Chuo-ku, Kobe 650-0047, Hyogo, Japan

A 72-year-old woman with dyspnoea and oedema was admitted to our hospital. She had cancer in her right breast in her 30s and had undergone mastectomy. She was disease-free thereafter until her recent symptoms. The electrocardiogram indicated an incomplete right bundle branch block, QT prolongation, and negative T-wave in leads V1–V3 (Supplementary material online, *Figure* *SA*). Transthoracic echocardiography (parasternal short-axis view) revealed right ventricular (RV) obstruction by a tumour growing from RV free wall to the inner cavity. We suspected intra-tumoural necrosis because of an anechoic region in the tumour (*Panel A*, *Videos 1* and 2). The peak blood flow velocity at the RV obstruction site was 4.6 m/s (*Panel B*). The wall motion of the right ventricle was reduced due to tumour infiltration. Contrast-enhanced computed tomography (CT) revealed a lobulated, enhancing mass occupied in the RV free wall (*Panel C*, *Video 3*). A small pericardial effusion was noted, with no echocardiographic features of tamponade. Moreover, we detected bilateral pleural effusion on the dominant right side. ^18^F-fluorodeoxyglucose (FDG) positron emission tomography/CT showed FDG accumulation in the RV tumour (*Panel D*). Cardiac magnetic resonance imaging showed a narrow RV cavity (arrows) due to the tumour position (*Panel E*, Supplementary material online, *Video S1*). We diagnosed metastasis originating in the RV free wall using multimodal imaging. Histopathological examination revealed a metastatic adenocarcinoma consistent with breast origin (*Panel F*). Immunohistochemical analysis was positive for CK AE1/AE3 and GATA3, and the final pathological diagnosis was breast cancer metastasis.

Supplementary material is available at *European Heart Journal - Case Reports* online.

**Consent:** The authors confirm that written consent for submission and publication of this case report including images and associated text has been obtained from the patient in line with COPE guidance.

**Conflict of interest:** None declared.

**Funding:** None declared.

**Panel:**
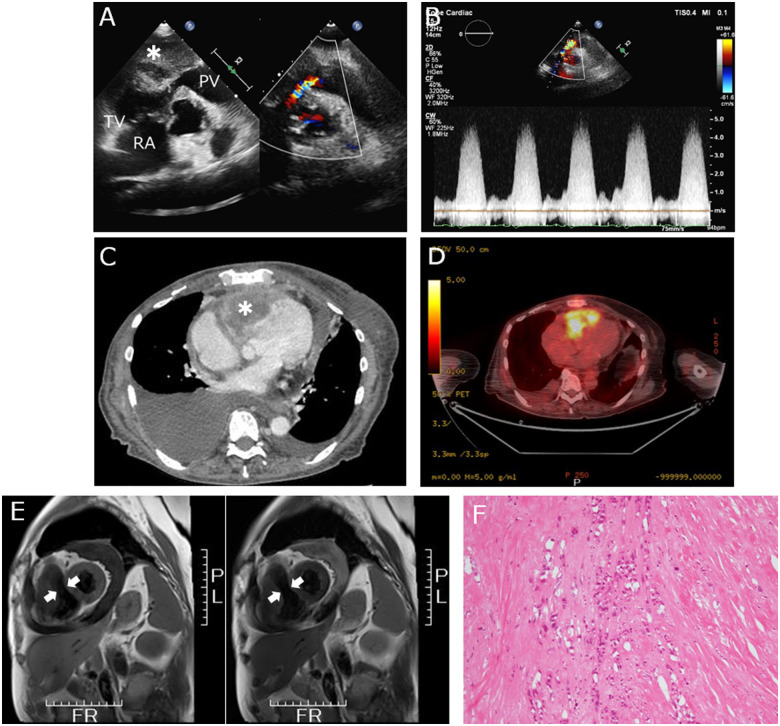
Final diagnosis confirmed by multimodal imaging and histopathological findings. Echocardiography showing RV obstruction and accelerated blood flow of 4.6 m/s was observed at the site of obstruction (*Panels A* and *B*). Contrast-enhanced CT showing lobulated enhancing mass (*) occupied in the RV free wall (*Panel C*). ^18^F-fluorodeoxyglucose positron emission tomography showed FDG accumulation in the RV free wall (*Panel D*). Magnetic resonance imaging showed narrowing of the right ventricle (arrows) due to the influence of the tumour (*Panel E*). Histopathological examination of the tissue samples derived from a thoracotomy RV myocardium biopsy showed a metastatic adenocarcinoma consistent with breast origin (*Panel F*). PV, pulmonary valve; RA, right atrium; TV, tricuspid valve.

## Supplementary Material

ytab290_Supplementary_Data

