# Supplementary figures and images for: Multimodal imaging of right ventricular obstruction due to metastatic cardiac tumour
Source: Eur Heart J Case Rep. 2021 Jul 24;5(7):ytab290. doi: 10.1093/ehjcr/ytab290 (PMC8343449; doi:10.1093/ehjcr/ytab290)

## Slide 1
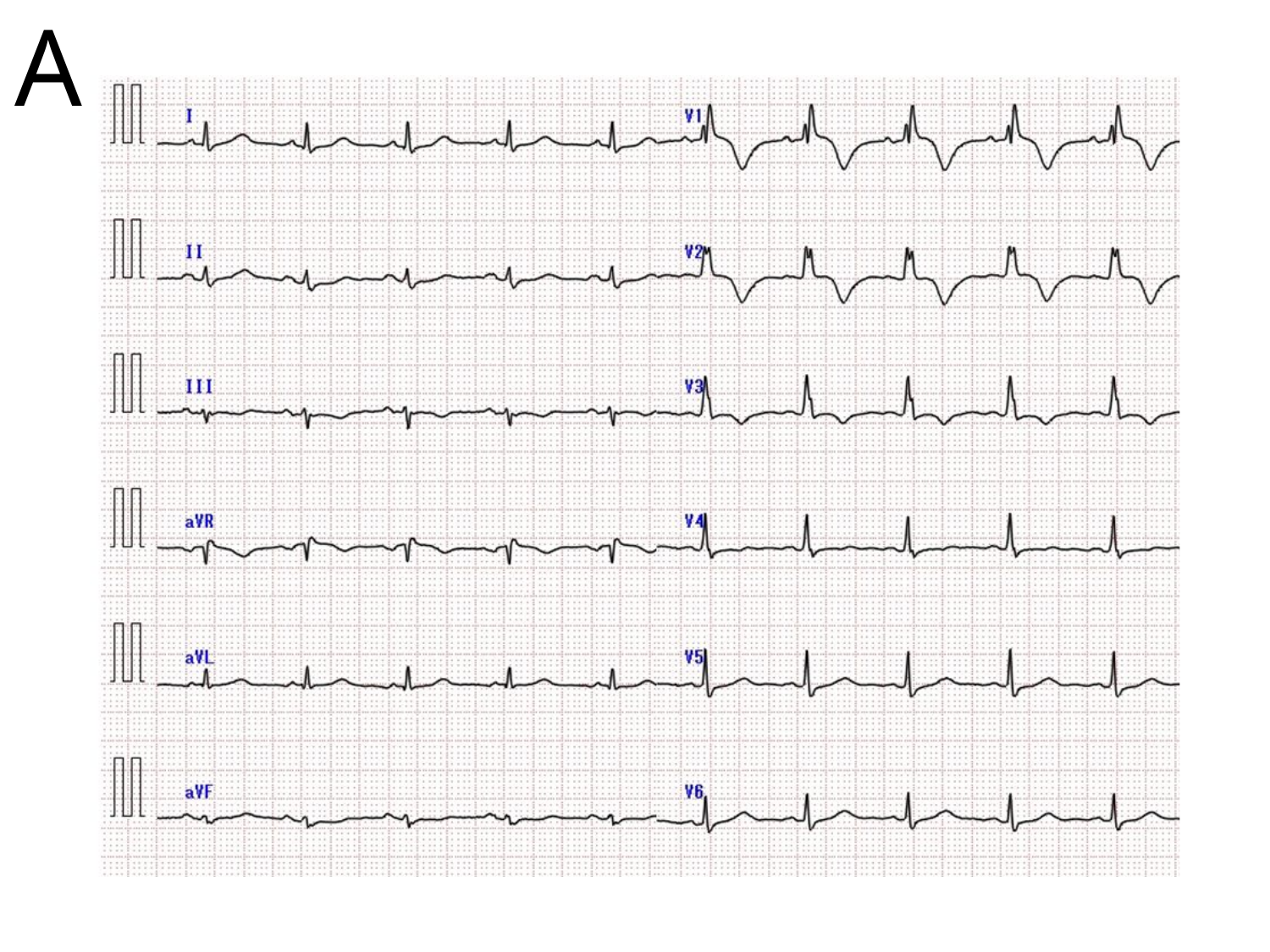

A

Supplement: ytab290_Supplementary_Data [file ytab290_Supplementary_Data.zip › Supplemental Figure.pptx]
